# ISG15 Modulates Development of the Erythroid Lineage

**DOI:** 10.1371/journal.pone.0026068

**Published:** 2011-10-12

**Authors:** Ana Leticia Maragno, Martine Pironin, Hélène Alcalde, Xiuli Cong, Klaus-Peter Knobeloch, Frederic Tangy, Dong-Er Zhang, Jacques Ghysdael, Christine Tran Quang

**Affiliations:** 1 CNRS (Centre National de la Recherche Scientifique) UMR3306, Orsay, France; 2 INSERM (Institut National de la Santé et de la Recherche Médicale) U1005, Orsay, France; 3 Institut Curie, Centre Universitaire, Bat 110 91405, Orsay, France; 4 Neuropathology, University Clinic Freiburg, Freiburg, Germany; 5 Unité de Génomique Virale et Vaccination, CNRS URA-3015, Institut Pasteur, Paris, France; 6 University of California San Diego, Moores University of California San Diego Cancer Center, La Jolla, California, United States of America; Southern Illinois University School of Medicine, United States of America

## Abstract

Activation of erythropoietin receptor allows erythroblasts to generate erythrocytes. In a search for genes that are up-regulated during this differentiation process, we have identified ISG15 as being induced during late erythroid differentiation. ISG15 belongs to the ubiquitin-like protein family and is covalently linked to target proteins by the enzymes of the ISGylation machinery. Using both *in vivo* and *in vitro* differentiating erythroblasts, we show that expression of ISG15 as well as the ISGylation process related enzymes Ube1L, UbcM8 and Herc6 are induced during erythroid differentiation. Loss of ISG15 in mice results in decreased number of BFU-E/CFU-E in bone marrow, concomitant with an increased number of these cells in the spleen of these animals. *ISG15^-/-^* bone marrow and spleen-derived erythroblasts show a less differentiated phenotype both *in vivo* and *in vitro*, and over-expression of ISG15 in erythroblasts is found to facilitate erythroid differentiation. Furthermore, we have shown that important players of erythroid development, such as STAT5, Globin, PLC γ and ERK2 are ISGylated in erythroid cells. This establishes a new role for ISG15, besides its well-characterized anti-viral functions, during erythroid differentiation.

## Introduction

Erythropoiesis is a tightly regulated process that allows the daily production of large numbers of circulating red blood cells. In adults, it mainly occurs in bone marrow but upon anemia induction, erythropoiesis can also shift to the spleen as “stress erythropoiesis” [1]. The earliest characterized erythroid progenitor is the BFU-E that can be identified by its ability to form large multifocal colonies in semi-solid medium. This progenitor then matures into more differentiated progenitors, CFU-E. From this stage, erythroblasts undergo further maturation that can be identified notably by morphological changes, sequentially giving rise to pro-erythroblasts, basophilic, polychromatic and orthochromatic erythroblasts that finally enucleate to generate reticulocytes. Several signaling pathways have been shown to regulate the balance between proliferation/differentiation and apoptosis of erythroid cells. *In vivo* and *in vitro* studies have highlighted the essential role of the erythropoietin receptor (EpoR) during terminal differentiation. *In vivo*, knockout of either the Epo or EpoR genes lead to embryonic death around E13.5, associated with anemia [2]. This phenotype is linked to the inability of committed CFU-E progenitors to terminally differentiate in erythrocytes. At the molecular level, EpoR is pre-associated with the protein tyrosine kinase JAK2, which becomes activated after Epo binding to its receptor. This results in the phosphorylation of tyrosine residues of the cytoplasmic tail of EpoR leading to the subsequent activation of intracellular signaling pathways including PI3K, ERK1/2 and STAT transcription factors [3]. Several STATs can be activated by EpoR which includes STAT1, STAT3 and STAT5 depending upon the experimental setting. Studies of genetically modified mice for either STAT1 or STAT5 have shown that these factors play an important role during erythropoiesis. Indeed, STAT5 a/b knockout mice suffer from anemia and die around birth [4], [5], [6]. Lack of STAT1 results in a general reduction of erythroid progenitors that are delayed in their terminal differentiation, accompanied by increased splenic stress erythropoisis [7].

In a search for new candidates which could be of importance to regulate erythroid differentiation, we have identified *ISG15* as an induced gene during this process. *ISG15* is one of the earliest genes induced upon interferon (IFN) type I (α/β) stimulation [8]. Study of its promoter has allowed to characterize the ISRE (Interferon-Stimulated Response Element), on which several transcription factors can bind, in particular the ISGF3 complex composed by STAT1, STAT2 and IRF9 [9]. ISG15 belongs to the ubiquitin-like protein family that also includes SUMO, Nedd8 and Fat10. Akin to the ubiquitylation process, ISG15 can be covalently linked to lysine residues of target proteins following a cascade of enzymatic reactions that involves an E1 activating enzyme (Ube1L), an E2 conjugating enzyme (UbcM8) and several E3 ligases (Herc6, EFP and HHARI) in a process named ISGylation. This process can be reversed and several proteases able to remove ISG15 have been identified, among them Usp18 [10]. Recently, ISGylation was shown to broadly target newly synthesized proteins in IFN-I-stimulated cells [11], yet for only a few number of proteins, the consequences of ISGylation have been elucidated [12], [13]. *ISG15* knockout mice are born viable and fertile without major developmental defect under steady state conditions [14]. However, and in line with the fact that *ISG15* is an interferon-inducible gene, these mice show an increased susceptibility to a variety of viruses [10].

We report here that: (i) ISG15 expression and protein ISGylation are induced during erythroid differentiation; (ii) ISG15 induction is mostly independent of IFN signaling and partially dependent upon activation of EpoR signaling; (iii) *ISG15^-/-^* erythroblasts have an intrinsic differentiation defect *in vitro*; (iv) mice lacking ISG15 show impaired erythropoiesis *in vivo*; (v) important players of erythroid development, including STAT5, Globin, PLCγ and ERK2 are ISGylated in erythroid cells.

## Results

### ISG15 expression and protein ISGylation are induced during erythroid differentiation

**Figure 1:**
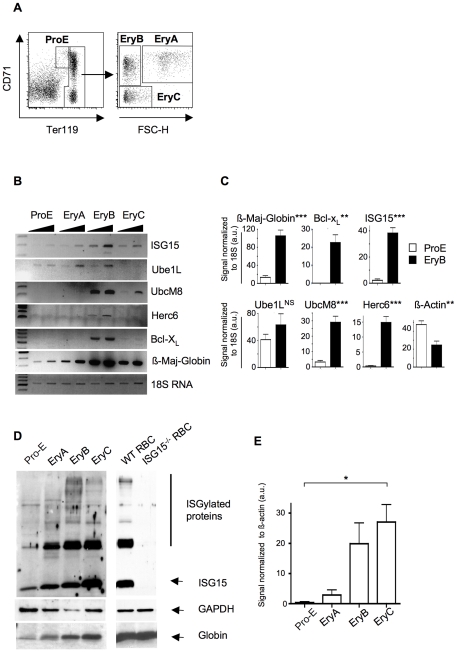
*ISG15* expression and protein ISGylation are induced during *in vivo* erythroid differentiation. (**A**) Sorting procedure of the different erythroblasts populations from bone marrow cells of WT Bl6/J mice. (**B**) RT-PCR analyses of *ISG15, UbcM8, Herc6, Ube1L, Bcl-X_L_* and *ß-Major-Globin* mRNA in sorted Pro-erythroblasts (Pro-E: large cells, CD71^high^, Ter119^med^), basophilic erythroblasts (EryA: large cells, CD71^high^, Ter119^high^), polychromatic (EryB: small cells, CD71^med^, ter119^high^) and orthochromatic erythroblasts/reticulocytes (EryC: small cells, CD71^low^, ter119^high^) as described in Mat & Met. Experiments were normalized to 18S Ribosomal RNA expression. (**C**) Statistical analysis of the induction of the expression of *ß-Maj-Globin, Bcl-X_L_, ISG15*, *Ube1L, UbcM8* and *Herc6 m*RNA. Quantification was performed as described in Mat & Met. Note the two-fold decreased expression of a second housekeeping gene, ß-actin during differentiation. au = arbitrary unit. (**D**) Western blot analyses of whole cell extracts of the indicated erythroid subpopulations using anti-ISG15 (top panels) antibody. Middle panels show GAPDH levels as loading control. Bottom panels show globin accumulation as detected with Ponceau staining of the membranes. Whole cell extracts were prepared from sorted wild-type bone marrow cells as in A or from RBC of WT and *ISG15^-/-^* mice. (**E**) Statistical analysis of the induction of ISG15 at the protein level during *in vivo* erythroid differentiation normalized to ß-Actin. Quantification was performed as described in Mat & Met. au = arbitrary unit.

To determine if ISG15 expression and protein ISGylation are induced during *in vivo* erythroid differentiation and if so, at which stage, mouse bone marrow (BM) cells were sorted according to cell surface expression of the transferrin receptor (CD71), glycophorin A-associated protein (Ter119) and size (FSC-H) (Figure 1A). These markers are conventionally used to sort immature pro-erythroblasts (Pro-E) from their progeny, namely basophilic erythroblasts (EryA), polychromatic erythroblasts (EryB) and orthochromatic erythroblasts/reticulocytes (EryC) [15]. RNA extracted from each sorted population was subjected to semi-quantitative RT-PCR analyses. *ISG15* transcript was expressed at low levels in ProE, increased in EryA and EryB and reduced in EryC (Figure 1B), thus presenting a kinetic of expression similar to that of *ß-Globin*, a gene induced upon erythroid differentiation. Expression of the ISG15 conjugation enzyme *UbcM8* and the E3 ligase *Herc6* was strongly induced in EryB, their expression profile resembling that of *Bcl-X_L_*, another gene known to be expressed late during erythroid differentiation [16]–[18]. Figure 1C shows that the expression of these genes is significantly up-regulated comparing ProE and EryB developmental stages, except for Ube1L. At the protein level, ISG15 and protein ISGylation were high in EryB and in EryC, as compared to ProE and EryA progenitors (Figure 1D and quantification of ISG15 expression in Figure 1E). ISG15 expression remained high in circulating red blood cells (RBC) (figure 1D). These results show that ISG15 protein expression and cellular protein ISGylation are up-regulated at the late stages of erythroid differentiation.

**Figure 2:**
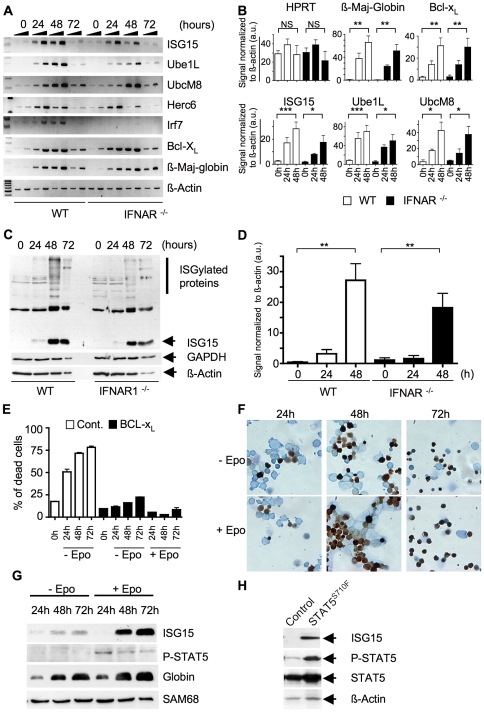
*ISG15* expression during erythroid differentiation is independent of IFN signaling and partially dependent upon Epo signaling. (**A**) semi-quantitative RT-PCR analyses of *ISG15, Ube1L, UbcM8, Herc6, Irf7, Bcl-x_L_* and *ß-Major-Globin* mRNA expression in WT or *IFNAR^-/-^* differentiating primary erythroblasts. Bone marrow erythroblasts of each genotype were maintained in proliferation conditions (SCF, Epo and Dex) for one week and next induced to differentiate in response to Epo alone for three days. Cells were collected every 24 hours as indicated and RNA extracted. The experiment was normalized to *ß-Actin* mRNA expression. (**B**) Statistical analysis of the induction of the expression of *ß-Maj-Globin, Bcl-X_L_*, *ISG15*, *Ube1L*, *UbcM8* and *Herc6* mRNA in differentiating WT and IFNAR^-/-^ erythroblasts. Quantification was performed as described in Mat & Met. Note the unchanged expression of a second housekeeping gene *HPRT*. au = arbitrary unit. (**C**) Whole cell protein extracts were prepared from WT or *IFNAR^-/-^* erythroblasts maintained as in A and analyzed on a 10% acrylamide gel for *ISG15* expression using an anti-ISG15 antibody. Anti-ß-Actin and anti-GAPDH were used as loading controls. (**D**) Statistical analysis of the induction of ISG15 during erythroid differentiation as normalized to ß-Actin. Quantification was performed as described in Mat & Met. au = arbitrary unit. (**E and F**) A p53^-/-^ erythroid cell line expressing exogenous hBcl-X_L_ was switched from proliferation conditions (Epo, SCF, Dex) to differentiating medium in the presence or absence of Epo. Cells were collected every 24 hours as indicated and analyzed for their ability to (e) survive as measured by propidium iodide staining in flow cytometry analyses; (f) differentiate as shown by analysis of their morphology after benzidine/May-Grunwald staining. Note the significant induction of cell death in control cells maintained in absence of Epo; in contrast, hBCL-xL erythroblasts are strongly protected from apoptosis. (**G**) Cells were lyzed according to cell number and volume. ISG15 expression was analyzed on a 15% acrylamide gel using anti-ISG15 antibody, activation of the EpoR/STAT5 signaling pathway was monitored using anti-P-STAT5 antibody, differentiation was monitored using anti-Globin antibody and loading control was performed using anti-SAM68 antibody. (**H**) Mock and mscv-puro-STAT5^S710F^ transduced p53^-/-^ erythroid cell line maintained under proliferation conditions were lyzed and analyzed for ISG15 expression using anti-ISG15 antibody on a 10% acrylamide gel (Top panel). P-STAT5 was detected at a higher level in mscv-puro-STAT5^S710F^ transduced cells while only a modest increase in the total amount of STAT5 can be noted. Anti-β -Actin was used as a loading control.

Because ISG15 and the genes encoding the enzymes involved in protein ISGylation were first characterized as IFN regulated genes [9], we next investigated whether IFN signaling was responsible for the induction of ISG15 expression and protein ISGylation during terminal erythroid differentiation. For this, we compared the expression of both ISG15 and of the components of the ISGylation machinery in differentiating wild-type and *IFNAR^-/-^* primary erythroblasts in which IFNα/β signaling is abrogated [19]. Primary cultures of proliferating erythroblasts can be expanded from bone marrow cells in the presence of Epo, Stem Cell Factor (SCF) and Dexamethasone (Dex) and can be induced to terminally differentiate within 3 days upon SCF and Dex removal and in the continuous presence of Epo [20]. Under these conditions, cells undergo 3 to 4 divisions accompanied with G1 phase shortening which ultimately results in cell size reduction, hemoglobin accumulation and finally enucleation. In the absence of Epo these erythroblasts rapidly die by apoptosis [20]. We found that *ISG15*, *Ube1L*, *UbcM8* and *Herc6* transcripts were up-regulated at the late stages of Epo-induced differentiation of primary wild-type erythroblasts (Figure 2A, WT panel and Figure 2B for quantification and statistical analyses). Their induction profile resembles that of *Bcl-X_L_* and *ß-Major-Globin* (Figure 2A). At the protein level, up-regulation of ISG15 expression was accompanied by protein ISGylation, as evidenced by the accumulation of ISG15 adducts to high molecular weight proteins (Figure 2C, WT panel). Similar results were obtained using a p53^-/-^ erythroid cell line [21] (data not shown and see thereafter). The induction kinetics of *ISG15, Ube1L, UbcM8* and *Herc6* was found unchanged in differentiating IFNAR1^-/-^ erythroblasts (Figure 2A) as compared to wild-type cells, although dampening of the overall expression level of these genes was observed (Figure 2A and 2B). In contrast, expression of *Irf7*, a bona fide IFNα/β responsive gene, was abrogated in IFNAR1^-/-^ differentiating erythroblasts (Figure 2A). At the protein level, ISG15 and ISGylation were also found induced in differentiating *IFNAR^-/-^* erythroblasts, although at somewhat reduced levels as compared to wild-type cells (Figure 2C and 2D). Similar results were found using primary erythroblasts from *IFNAR/IFNGR* double-deficient mice (data not shown). These data show that ISG15 upregulation and ensuing protein ISGylation are mostly independent of IFNα/β and IFNγ signaling in differentiating erythroblasts.

EpoR signaling is an absolute prerequisite for the differentiation of CFU-E cells [2]. We thus analyzed whether ISG15 expression could be regulated through this signaling pathway. Since erythroblasts rapidly die by apoptosis when deprived from Epo, we relied upon previous observations that enforced expression of anti-apoptotic members of the BCL-2 family can rescue this cell death phenotype and allows their terminal differentiation in absence of Epo [22], [23]. In the absence of Epo, exogenously expressed hBCL-X_L_ allowed survival and terminal differentiation of erythroblasts as analyzed by flow cytometry (Figure 2E), morphological analysis (Figure 2F) and western blot analysis of globin accumulation (Figure 2G). Under this condition, expression of ISG15 was slightly induced but was clearly weaker as compared to parallel culture of hBCL-x_L_-expressing erythroblasts differentiated in the presence of Epo (Figure 2G). This suggests that activation of the EpoR signaling pathway may participate to ISG15 induction. Furthermore, as shown in Figure 2H, the level of ISG15 was also found induced in proliferating, undifferentiated erythroblasts expressing a constitutively activated form of STAT5 (STAT5^S710F^), an essential effector of EpoR signaling in erythroid development [4]–[6]. Although acute Epo stimulation does not immediately induce ISG15 expression (our unpublished observations), these data suggest that ISG15 expression during erythroid terminal differentiation is at least partially dependent upon EpoR signaling.

### *ISG15^-/-^* erythroblasts show an impaired ability to differentiate *ex vivo*

**Figure 3:**
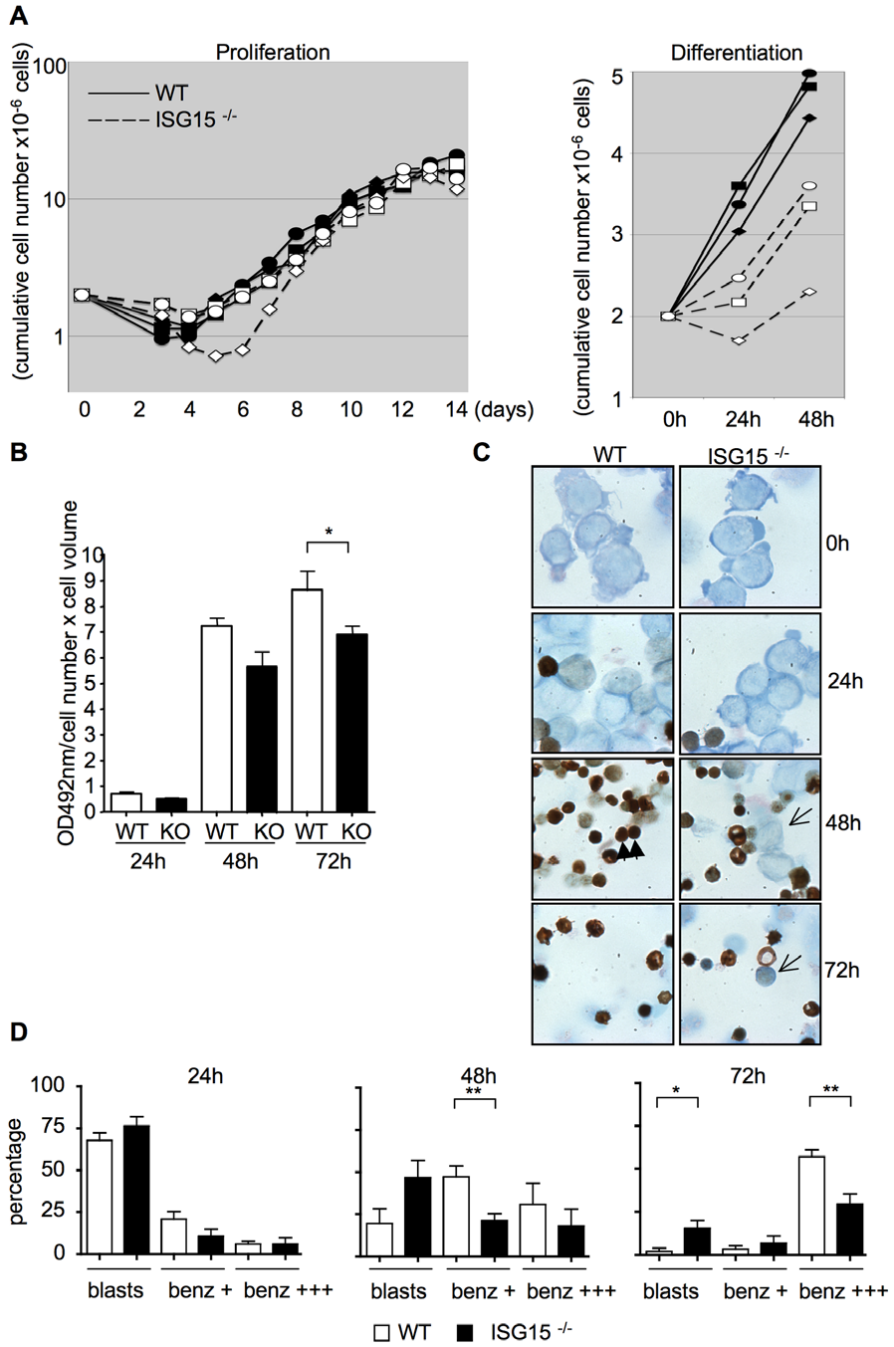
*ISG15* deficiency impairs *in vitro*-induced erythroid differentiation. (**A**) Erythroblasts from the BM of either wild-type or *ISG15^-/-^* mice were maintained under proliferation conditions (SCF, Epo, Dex) and switched to Epo alone to induce differentiation. Cumulative cell number of three independent wild-type (black symbols) and *ISG15^-/-^* erythroblasts cultures (open symbols) are shown both under proliferation and differentiation conditions. Cells were counted with an electronic counter (CASY Scharfe). (**B**) Quantitative determination of hemoglobin contents of differentiating WT and *ISG15^-/-^* erythroblasts 24, 48 and 72 hours after differentiation induction. Normalized values (hemoglobin level per 10^6^ live cells) are shown. (**C**) Cytocentrifugation analyses followed by Benzidine-May-Grunwald staining of cells maintained either under proliferation conditions (day 0) or induced to differentiate in response to Epo. Differentiating cells are stained in brown by Benzidine (black arrow), immature eryhroblasts stain in blue. Note the presence of a significant proportion of immature cells in *ISG15^-/-^* culture (open arrow) as compared to WT culture. Representative fields are shown. (**D**) Quantification of cells of increasing maturity 24 h, 48 h and 72 h after the cells had been induced to differentitate. Cells (≥200) were counted per slide and mean values ±s.d. calculated from at least three independent experiments.

To address the importance of ISG15 expression for erythroid differentiation, we compared expansion and differentiation of erythroblast primary cultures derived from wild-type and *ISG15^-/-^* mice. We observed no major difference in expansion kinetics between WT and *ISG15^-/-^* erythroblasts cultures (Figure 3A, left panel). However, when these cultures were switched to differentiation conditions, cumulative cell numbers observed 2 and 3 days after differentiation induction were lower in *ISG15^-/-^* cultures as compared to wild-type erythroblasts (Figure 3A, right panel). This was accompanied by a lower level of hemoglobin accumulation in *ISG15^-/-^* erythroblasts, as analyzed by a colorimetric staining for Hb (Figure 3B) and by the reduced proportion of hemoglobin-positive cells in cytocentrifugation analyses (Figure 3C and 3D). We also observed the subsistence of immature erythroblasts in *ISG15^-/-^* cultures 2 days after differentiation induction (Figure 3C and quantification in Figure 3D). These results thus show that ISG15 deficiency intrinsically interferes with terminal erythroid differentiation.

**Figure 4:**
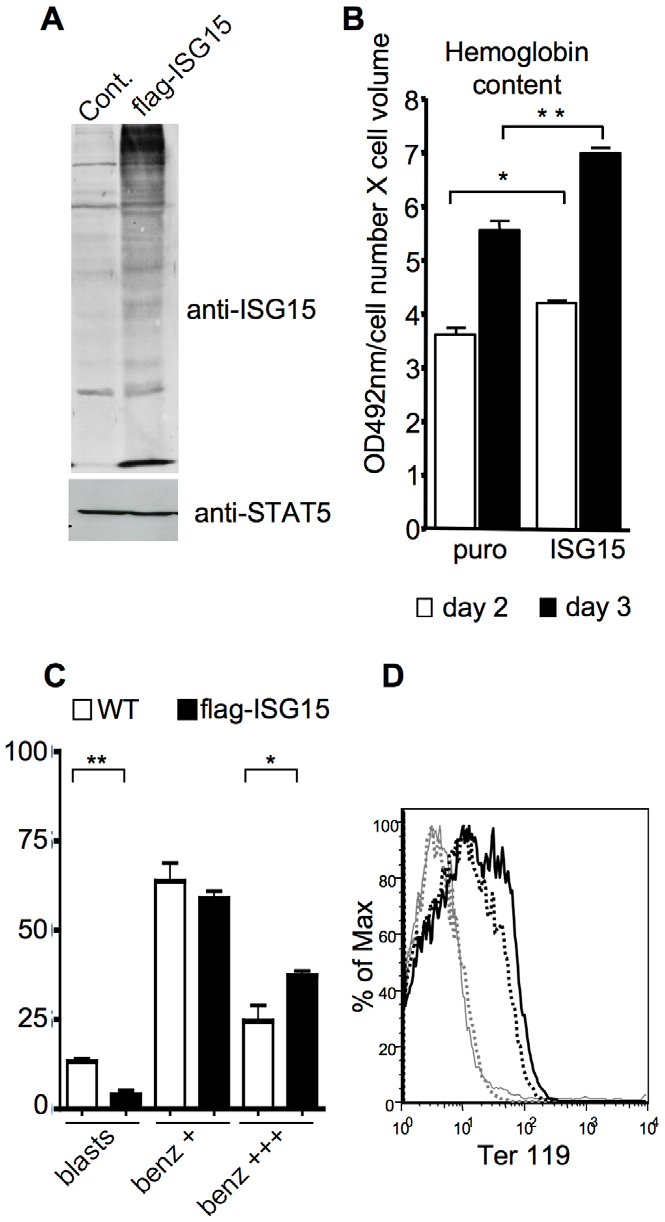
Enforced ISG15 expression facilitates erythroid terminal differentiation. (**A**) Western blot analysis of whole cell extract obtained from proliferating p53^-/-^ erythroid cell line transduced with either the control MSCV-puro, or MSCV-puro-ISG15-flag retroviruses. An anti-ISG15 antibody was used to show ISG15 expression and induction of protein ISGylation on a 10% acrylamide gel while anti-STAT5 was used as a loading control. (**B**) Hemoglobin content quantification analyses 48 and 72 hours after differentiation induction. (**C**) Quantification of cytospin preparation of 48 h-differentiating control and ISG15-overexpressing erythroblasts. Cells (≥200) were counted per slide and mean values ±s.d. calculated from at least three independent experiments (**D**) Flow cytometry analysis of erythroid cell surface marker Ter119, 72 hours after differentiation induction. Grey line: isotypic control, grey dashed line: control proliferating erythroblasts (1,34% Ter119 positive cells), black dashed line: control differentiating erythroblasts (24,2% Ter119 positive cells); black line: ISG15-differentiating erythroblasts (34,4% Ter119 positive cells).

In the reverse experiment, the p53^-/-^ erythroid cell line was transduced with a retroviral vector expressing Flag-tagged ISG15 (Figure 4A). We observed that expression of ISG15 in p53^-/-^ erythroid cell line was accompanied by an increase in protein ISGylation even under proliferative conditions (Figure 4A). When switched to differentiation conditions, ISG15 expressing erythroblasts showed an improved ability to differentiate as evidenced by a higher hemoglobin accumulation per live cell (Figure 4B), an increased proportion of hemoglobinized, benzidine-positive cells (Figure 4C), and a higher level of Ter119 expression as compared to controls (Figure 4D). Taken together, these data show that ISG15, the expression of which is increased during erythroid differentiation, facilitates the transition of differentiating cells through the late stages of erythroid maturation.

### *ISG15^-/-^* mice show an altered distribution of erythroid progenitors in bone marrow and spleen

To investigate the importance of ISG15 in erythropoiesis *in vivo*, we compared erythroblasts maturation in bone marrow and spleen of wild-type and *ISG15^-/-^* mice. We observed a decreased proportion of EryC, accompanied by an increased proportion of EryB in *ISG15^-/-^* bone marrow cells as compared to wild-type mice (Table 1B, bone marrow). This reduction of EryC population in *ISG15^-/-^* erythroblasts did not result from impaired survival of these cells or their progenitors as no increase in annexin V-positive cells was observed in any of the erythroblasts subpopulations from *ISG15^-/-^* bone marrow cells. Rather, *ISG15^-/-^* erythroblasts showed a reduction in apoptosis as compared to matched wild-type cells (data not shown). These results rather favor the notion of a requirement for ISG15 for the transition of polychromatic erythroblasts to the orthochromatic/reticulocyte stage.

**Table 1:** Altered distribution of erythroid progenitors in *ISG15^-/-^* bone marrow and splenic cells.

	WT	ISG15^-/-^	Pvalue	Sign
**A. Blood parameters (N = 14)**				
RBC count. M/mm3	10.90 +/- 0.14	10.71 +/- 0.13	0.338	ns
HGB level. g/dL	19.94 +/- 0.26	19.97 +/- 0.22	0.916	ns
MCH. pg	18.29 +/- 0.13	18.66 +/- 0.15	0.073	ns
MCHC. g/dL	39.29 +/- 0.28	39.24 +/- 0.21	0.889	ns
HCT. %	50.74 +/- 0.62	50.90 +/- 0.58	0.849	ns
MCV. fL	46.58 +/- 0.28	47.56 +/- 0.36	0.041	*
RDW. %	16.41 +/- 0.007	16.12 +/- 0.008	0.014	*
Retic count. %	2.75 +/- 0.20	5.03 +/- 0.49	0.0008	***
PLT count. m/mm3	972 +/- 61.92	830 +/- 88.43	0.199	ns
WBC count m/mm3	12.08 +/- 0.84	13.59 +/-1.03	0.265	ns
MONO. %	2.346 +/- 0.06	2.615 +/- 0.13	0.074	ns
LYMP. %	87.83 +/- 0.72	84.91 +/- 1.38	0.072	ns
GR. %	9.864 +/- 0.68	12.37 +/- 1.26	0.091	ns
**B. Flow cytometry analysis (N = 13)**				
**Bone marrow**				
Ter119+	47 +/- 1.1	46 +/- 1.1	0.6816	ns
ProE	1.0 +/-0.008	1.3 +/- 0.11	0.0475	*
EryA	16.90 +/- 0.55	17.42 +/- 0.66	0.5554	ns
EryB	21.67 +/- 0.62	26.89 +/- 1.87	0.0141	*
EryC	57.50 +/- 0.71	51.58 +/- 1.94	0.0086	**
**Spleen**				
Ter119+	66 +/- 2.3	56 +/- 2.1	0.0045	**
ProE	0.06 +/- 0.01	0.15 +/- 0.04	0.0427	*
EryA	0.90 +/-0.17	2.14 +/- 0.36	0.0087	**
EryB	1.70 +/- 0.17	2.48 +/- 0.26	0.0277	*
EryC	93.91 +/- 0.50	90.52 +/- 0.68	0.0008	***
**C. Colony assays (N = 11)**				
**Bone marrow**				
BFU-E	57.20 +/- 5.28	38.70 +/- 5.25	0.023	*
CFU-E	413.4 +/- 26.65	320.8 +/- 31.02	0.033	*
**Spleen**				
BFU-E	10.31 +/- 1.68	17.62 +/- 2.13	0.013	*
CFU-E	22.54 +/- 2.71	61.23 +/- 5.90	P<0.0001	***
Spl. Weight. g	0.083 +/- 0.002	0.100 +/- 0.003	0.0006	***

(**A**) Blood was collected from mice at 8–10 weeks of age. Hematologic measurements were performed on a MS9 Hematology Analyzer (MELET SCHLOESING Laboratoires). The data are mean +/- SEM (N = 14). RBC indicates red blood cells; HGB, hemoglobin; MCH, mean corpuscular hemoglobin; MCHC, MCH concentration (calculated); HCT, hematocrit; MCV, mean corpuscular volume; RDW, RBC distribution width, Retic, reticulocytes; PLT, platelets; WBC, white blood cells; MONO, monocytes, LYMP, lymphocytes and GR, granuloctes. (**B**) Quantitative analysis of the distribution of the different erythroblasts subsets in age-matched WT versus *ISG15^-/-^* mice. Flow cytometry analyses using the cell surface markers CD71 and Ter119 of bone marrow and spleen cells isolated from WT or *ISG15^-/-^* mice (as described in Figure 1A). Dead cells (7AAD^+^) were excluded from the analysis. The data are mean +/- SEM (n = 13). (**C**) 2.10^5^ BM and 2.10^6^ spleen cells from mice at 8–10 weeks of age were used to assay BFU-E and CFU-E numbers in MethoCult M3334 (StemCell Technologies). For CFU-E assays, colonies were counted at day 2 and for BFU-E assay, at day 4. The data are mean +/- SEM (n = 11).

Although blood erythrocytes numbers and hematocrit level were similar in *ISG15^-/-^* and wild-type mice (Table 1A), a slight modification of erythrocytes properties (cell volume and width distribution, Table 1A) was observed. Moreover the reticulocyte numbers were doubled in ISG15^-/-^ mice. This suggested that *ISG15^-/-^* mice may develop compensatory stress erythropoiesis. To address this question, we compared the number of BFU-E and CFU-E from wild-type and *ISG15^-/-^* mice in both bone marrow and spleen. *ISG15^-/-^* bone marrow contained a reduced number of BFU-E and CFU-E as compared to wild-type bone marrow (Table 1C). This decrease in bone marrow BFU-E/CFU-E was associated with an increase in splenic BFU-E/CFU-E (Table 1C, spleen) and a slight but significant increase of *ISG15^-/-^* spleen weight as compared to wild-type mice (Table 1C). These results show that early bone marrow erythropoiesis is inhibited in *ISG15^-/-^* mice and is accompanied by a compensatory increase in splenic erythropoiesis. Of note, terminal erythroid differentiation was also altered in splenic erythroblasts. Indeed, a significant decrease in the EryC fraction was observed in *ISG15^-/-^* splenic cells as compared to wild-type, with a concomitant increase in the proportion of ProE, EryA and EryB erythroblasts (see Table 1B, Spleen). We next analyzed the response of *ISG15^-/-^* mice to stress-induced erythropoiesis. Upon phenylhydrazine-induced hemolytic anemia, the percentage of reticulocytes in peripheral blood was increased in *ISG15^-/-^* mice 3 days after the start of the treatment, but reached the same level as that observed in wild-type mice afterwards. However, this faster response did not lead to accelerated recovery from anemia in *ISG15^-/^* ^-^ mice (data not shown), a phenotype reminiscent to that described in STAT1-deficient mice [7]. Finally, as the level of ISG15 protein was high in erythrocytes (Figure 1B), the consequences of its absence on the lifespan of RBC was analyzed. No major difference could be noticed between wild-type and *ISG15^-/-^* RBC lifespan (data not shown).

### PLCγ, ERK2, Globin and STAT5 transcription factors are ISGylated in erythroid cells

**Figure 5:**
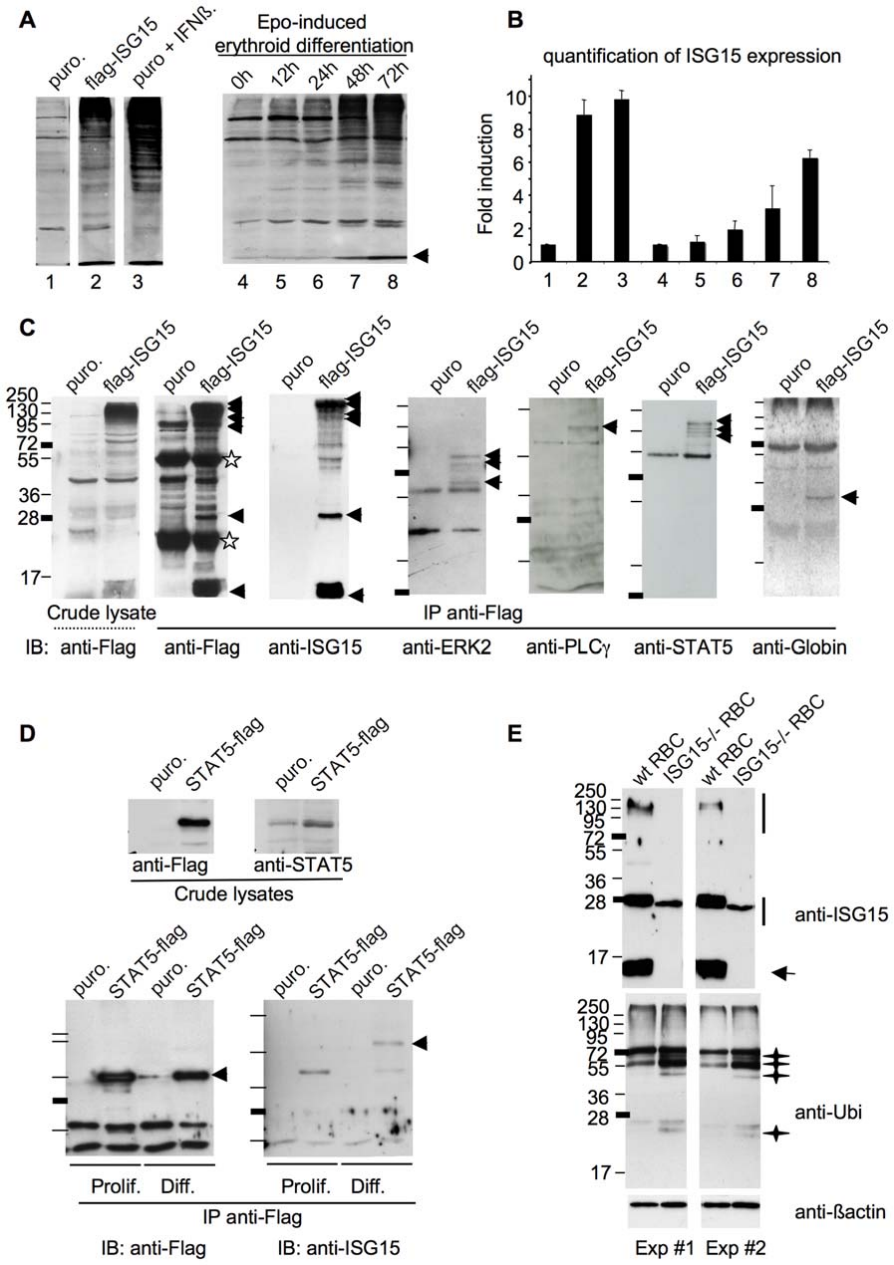
Identification of several ISGylation substrates in erythroid cells and increased ubiquitination in *ISG15^-/-^* RBCs. (**A**) Proliferating erythroblasts were left either untreated (1), or stimulated with IFNß (100 U/ml) for 24 h (3), or induced to differentiate for 72 h (lane 4 to 8). Cells were lyzed according to size and number and ISG15/ISGylation level was compared by western blotting to the level of ISG15/ISGylation present in proliferating Flag-ISG15-expressing p53^-/-^ erythroid cell line. The arrow points to ISG15 band (**B**) Quantification of ISG15 level in independent western blot experiments. (**C**) Proliferating control and Flag-ISG15-expressing p53^-/-^ erythroid cell line were lysed and ISGylated proteins searched after a Flag immunoprecipitation. Anti-Flag detect ISG15-Flag and ISGylated proteins (arrow heads) in the crude lysates (dashed line) and are found enriched after the immunoprecipitation (plain line bar). Stars point to light and heavy chains of immunoglobulin. For the detection of ISGylated Globins, cells were induced to differentiate for 72 hours in order to induce globin expression. ISGylated proteins are indicated by arrowheads. Normal unmodified molecular weight of the proteins are: ERK2 (42kDa), PLCγ (150kDa), STAT5 (90kDa), Globin (13kDa). For ERK2 and STAT5 detection, cell lysates were run on a 10% acrylamide gel, for ISG15 and Globins detection on a 15% acrylamide gel and for PLCγ on a 8% gel. (**D**) Proliferating or 48 hours-differentiating control and STAT5-Flag-expressing p53^-/-^ cell line were lysed and analyzed for exogenous STAT5 expression either using a Flag (upper left panel) or a STAT5 antibody (upper right panel). Note the modest increase in the total amount of STAT5 induced by the expression of STAT5-Flag. ISGylated STAT5 was searched after a Flag immunoprecipitation followed by either a Flag (bottom left panel) or an ISG15 western blot analyses (bottom right panel). Extracts were run on a 7% acrylamide gel. (**C**) Western blot analysis of RBC extracts from WT and *ISG15^-/-^* mice using a 15% acylamide gel. ISG15 expression and ISGylation were analysed using anti-ISG15 antibody (top panel), ubiquitination was monitored using anti-ubi antibody (intermediate panel) and anti-β-Actin was used as a loading control.

We next investigated the nature of cellular proteins ISGylated in erythroblasts. For this, we used the flag-ISG15-expressing p53^-/-^ erythroid cell line. These cells show an expression level of ISG15 comparable to that obtained in response to IFNß stimulation, a physiological inducer of ISG15 (Figure 5A and quantification in Figure 5B). Besides, this exogenous level of ISG15 was found only 2 fold higher than the level of ISG15 detected in terminally differentiating erythroblasts (Figure 5A and 5B, compare lane 2 to lane 8). ISGylated proteins were purified by a flag immunoprecipitation from either control or flag-ISG15-expressing erythroblasts (Figure 5C, left panel). Western blot analysis of these immunoprecipitates using either an anti-Flag or an anti-ISG15 antibody revealed, besides ISG15 itself, several ISGylated proteins (arrows in Figure 5C). Western blot analyses of the same immunoprecipitates using antibodies specific to ERK2, PLCγ, STAT5 and Globin identified higher molecular weight adducts only in flag-ISG15 precipitates (Figure 5C, arrows). This shows that endogenous ERK2, PLCγ, STAT5 and Globin can be ISGylated in erythroblasts, the proportion of ISGylated protein never exceeding 4 to 5% of total protein for STAT5 for instance. While enforced ISG15 expression could lead to unspecific ISGylation of a broad spectrum of proteins, we found several other proteins not being ISGylated under the same experimental setting (data not shown). Furthermore, when p53^-/-^ erythroid cell line engineered to express a STAT5-flag protein was analyzed for the status of STAT5 ISGylation using the same immunoprecipitation protocol as in Figure 5C, ISGylated STAT5 was detected in differentiating cells but not in proliferating erythroblasts (Figure 5D). This showed that STAT5 is also ISGylated by endogenous levels of ISG15 during erythroid differentiation.

### Ubiquitination is increased in *ISG15^-/-^* RBC

A cross-talk between ISG15 and ubiquitination has been previously suggested [12], [24], [25]. We thus sought to investigate whether absence of ISG15 could alter the ubiquination profile of erythroid cells. For this, RBC from wild-type and *ISG15^-/-^* mice were collected and cell lysates performed in the presence of NEM to prevent degradation of ubiquitinated proteins. ISGylation and ubiquitination were examined by Western blot analysis. High levels of ISG15 as well as ISGylated cellular proteins were observed in RBC but not in *ISG15^-/-^* RBC (Figure 5E, top panels). In *ISG15^-/-^* RBC, the anti-ubiquitin antibody recognized several additional bands that were weaker or absent in wild-type RBC lysates (Figure 5E, middle panels). These results suggest that a mechanism of ISG15 action in erythroid cells could be its interference with the ubiquitination pathway.

## Discussion

The results presented in this study demonstrate that the expression of ISG15 is physiologically up-regulated during late stages of erythropoiesis, show a consistent role for ISG15 in *in vivo* and *in vitro* erythroid differentiation and identify new targets of ISG15 in the erythroid lineage. ISG15 is one of the earliest genes shown to be induced upon type I IFN (α/β) stimulation [8]. In this study we show that ISG15 up-regulation is mostly independent of IFN signaling in the erythroid lineage. Indeed, in both *IFNAR1^-/-^* and *IFNAR1^-/-^/IFNGR^-/-^* double deficient erythroblasts, up-regulation of *ISG15, Ube1L, UbcM8* and *Herc6* transcripts is maintained during erythroid differentiation, whereas the expression of *Irf7*, a bona fide IFN-stimulated gene, is suppressed as expected. This suggests that in differentiating erythroblasts, IFN plays a minor role in the up-regulation of ISG15 and of the ISGylation machinery enzymes and implies that other signaling pathways regulate the expression of these genes in erythroid cells. Using erythroid cell culture models, we show that activation of the EpoR signaling pathway participates to ISG15 and ISGylation up-regulation. Indeed, Bcl-X_L_ expressing erythroblasts, that can differentiate either in the absence or presence of Epo [22], show a stronger up-regulation of ISG15 and protein ISGylation when differentiated in the presence of Epo as compared to cells differentiated in the absence of Epo. Besides, expression of a constitutively active mutant of STAT5 in proliferating, undifferentiated erythroblasts was sufficient to induce ISG15 and ISGylation. It is interesting to note that, although *ISG15* is not an immediate early gene induced in response to Epo (our unpublished observation), its expression closely follows that of *BCL-x_L_*, another indirect target of the EpoR/STAT5 axis [17], [26].

Our studies of *ISG15*-deficient mouse show that ISG15 plays a facilitating role in erythroid differentiation. Indeed, *ISG15^-/-^* primary erythroblasts present an impaired ability to terminally differentiate *in vitro* as shown by the decreased accumulation of erythrocytes and the persistence of immature cells in *ISG15^-/-^* cultures at the late stages the differentiation process, as compared to wild-type cultures. Although we cannot exclude a role of ISG15 on the erythroid stromal microenvironment, these observations indicate that *ISG15^-/-^* erythroblasts have an intrinsic defect at the late stages of differentiation. *In vivo*, *ISG15^-/-^* mice show decreased number of bone-marrow-derived BFU-E and CFU-E with a concomitant increase in the number of these progenitors in the spleen. This phenotype is reminiscent of stress erythropoiesis, which could result from the impaired erythroid terminal differentiation observed in *ISG15^-/-^* mice. Indeed, a smaller proportion of orthochromatic erythroblasts/reticulocytes accompanied by an increased proportion of polychromatic erythroblasts was observed in *ISG15^-/-^* mice as compared to wild-type mice, a phenotype observed in the erythroid compartment of both the bone marrow and spleen of *ISG15^-/-^* animals. However, this altered erythroid differentiation is not due to increased apoptosis of *ISG15^-/-^* early erythroblasts, as observed in either *STAT5*- or *STAT1*-deficient mice [5], [7], [27]. Instead, a decreased level of apoptosis was observed in all *ISG15^-/-^* erythroblast populations (data not shown). This phenotype is reminiscent to that observed, for example, in *Nix*-deficient mice. Lack of Nix, a pro-apoptotic protein also induced at the last stages of erythroid differentiation [28], [29], leads to a decrease in apoptosis and a defect in terminal erythroid differentiation [30]. As the importance of pro-apoptotic proteins in erythroid differentiation has been previously demonstrated [31], [32], one can hypothesize that ISG15, which is normally induced by IFN (a known inducer of apoptosis) could participate as a pro-apoptotic protein important for erythroid differentiation.

At the molecular level, it has been shown that ISG15 can act at least by three distinct mechanisms: (i) ISG15 can play a role of a cytokine; (ii) it can modulate the activity of specific target proteins via ISGylation; (iii) it can modulate the activity of proteins via non-covalent interaction. ISG15 has been purified as a RBC-derived neutrophil chemotactic factor from Plasmodium-yoelii infected mice [33]. We found high level of ISG15 in circulating RBC. It is thus tempting to speculate for a role of ISG15 in the immune defense against Plasmodium following its release from infected RBC and its ability to mobilize neutrophils. Only the comparison of the immune response of wild-type mice and mice deficient for *ISG15* in the erythroid lineage will allow to investigate the contribution of ISG15 in RBC to plasmodium infection resistance.

A number of proteins have been reported to be modified by ISGylation. The functional consequences of this post-translational modification could be determined for only a restricted number of these targets. For instance, ISGylation of the transcription factor IRF3 and the cap structure-binding protein 4EHP positively regulates the activity of these proteins [13], [34] while ISGylation inhibits the activity of other proteins, like CHMP5, a protein important for vesicular transport [35]. We report the identification of four ISGylation targets in erythroid cells, namely PLCγ, ERK2, STAT5 and globin. While PLCγ and ERK2 were previously identified as targets of ISG15, STAT5 and Globin represent novel substrates for ISGylation. These proteins have been shown to play essential roles in the erythroid lineage [5], [36], [37]. Thus, identification of the functional consequences of ISGylation of these targets may allow to better understand the role of ISG15 in erythroid differentiation.

Finally, ISG15 itself has been described to play a role in the regulation of specific target proteins, independently of the ISGylation process. For example, ISG15 inhibits virus budding via its ability to interact with the E3 ubiquitin ligase Nedd4 and thus prevent the ubiquitination of viral proteins [12], [25]. Additional experimental evidence suggests a cross-talk between ISG15 and the ubiquitination pathway. For instance, the level of polyubiquitinated proteins is increased in response to *ISG15* knockdown in ZR-75-1 breast cancer cells [24], which could result from a competition between ISG15 and ubiquitin for common E2 enzymes, such as UbcH8 [38], [39], and UbcH6 [40]. As (i) several essential proteins for the erythroid lineage have been shown to be regulated via their ubiquitination [41]–[43] and (ii) protein degradation via the proteasome is important for reticulocyte maturation [44], interference of ubiquitination by ISG15 could represent an alternative molecular mechanism to modulate erythroid differentiation. In line with this, we have noticed that *ISG15^-/-^* RBCs indeed show an increase in some ubiquitinated proteins, thus showing that ISG15 could modulate protein ubiquitination in erythroid cells. Taken together, our results suggest that ISG15 plays an important role in erythroid differentiation, but that the molecular cues underlying this phenotype may rely on ISGylation dependent and independent mechanisms.

## Materials and Methods

### Mice

*ISG15^-/-^* BL6 and their control littermates [14] and *IFNAR^-/-^* 129Sv mice and their control littermates [19] were maintained in specific-pathogen-free conditions at the animal facility of the Curie Institute (Orsay, France) and Pasteur Institute (Paris, France) respectively. Genotyping of *ISG15^-/-^* mice was done by PCR analysis of tail DNA. The primers used were: WT forward ^5′^GCCCCCATCCAGAGCCAGTGTT^3′^, WT/KO reverse ^5′^AGCCCCGATGAGGATGAGGTGT^3′^ and KO forward ^5′^CGCGAAGGGGCCACCAAAGAA^3′^. All experimental procedures were performed in accordance with the recommendations of the European Community (86/609/EEC) and the French National Committee (87/848) for the care and use of laboratory animals. All animal experiments were carried out under the supervision of J.G., who was authorized by the director of the Veterinary Services of the Prefecture de l'Essonne (agreement number 91-7). Animal care and use for this study were specifically approved by the ethics committee of the Curie Institute in compliance with the institutional guidelines.

### DNA plasmid constructs

Generation of pMSCV puro-flag-ISG15 was performed by cloning the amplified flag-ISG15 fragment using as template the pFlag-CMV-ISG17 plasmid and the primers: (Forward: ^5′^CCAGATCTGCCACCATGGACTACAAAGACGATGACG^3′^; Reverse: ^5′^CCTGGAATTCTTAGGCACACTGGTCCCCTCC^3′^). The PCR product was cloned into the EcoRI/BglII-digested pCR2.1 Topo plasmid (TOPO TA Cloning kit, Invitrogen) and sequenced. The EcoRI/BglII fragment was then cloned into the BglII/EcoRI –digested pMSCV-puro vector.

### Cell culture and retroviral transduction

PlatE ecotropic packaging cell line [45] were cultured in DMEM containing 10% FBS, 100 U/ml penicillin and 100 µg/ml streptomycin and transfected using Ca_3_(PO_4_)_2_ co-precipitation method with 40 µg/ml of pMSCV-puro-based retroviral constructs (Clonetech). Twenty-four hours after transfection, medium was changed and 24 hours later medium was collected as retroviral stocks. Primary erythroid progenitors and p53^-/-^ immortalized erythroid cell line (derived from fetal liver cells of p53^-/-^ mice) were cultured in serum-free medium (StemPro 34 plus nutrient supplement, Invitrogen) supplemented with 1 U/ml of human recombinant Erythropoietin (Jansen Cilag), 100 ng/mL of murine recombinant SCF (Peprotech) and 1 µM of Dexamethasone (Sigma). For differentiation induction, erythroblasts were washed twice with PBS and cultured in StemPro medium supplemented with 2 U/mL Epo and 0,5 mg/mL human holo-transferrin (Sigma). Cell numbers and size/volume were determined every day using an electronic cell counter (CASY-1, Scharfe-System) and cell density was maintained at 2–4×10^6^ cells/ml. Differentiated cells were analysed for hemoglobin content, cell size/volume and morphology as previously described [46]. For quantification of maturing cells, fields of cytospin preparation of three independent cultures were counted (>200 cells). Large, blue and Benzidine-negative cells were counted as erythroblasts, lightly benzidine stained cells as differentiating cells (Benz +) and dark, small cells as differentiated cells (Benz +++). Retroviral transduction of erythroblasts was performed by centrifuging 4×10^6^ erythroblasts, with 5 ml of platE supernatant containing the retroviral particules of interest and 6 µg/ml of polybrene for 2 hours, at 3000 rpm at 32°C, cultured as described and 48 hours post-infection, transduced cells selected in the presence of 1 mg/ml of puromycin (Sigma).

### Semi-quantitative RT-PCR analysis

Total RNA was isolated from 3.10^6^ cells using Trizol reagent (Invitrogen). 0,5 to 1 µg of RNA was reverse transcripted (RT)-PCR using random primers and the kit ImProm II Reverse Transcription System (Promega Corporation) according to the manufacturer's instructions. PCR were performed using two increasing doses (1∶2) of cDNA as indicated by the increment sign in the figures. GOTaq DNA Polymerase (Promega Corporation) and the following conditions were used: 94°C for 5 min, followed by n cycles at 94°C for 1 min, 58°C for 1 min, and 72°C for 1 min. The sequence of the amplimers were as follows: *ISG15*, forward 5′CCAGTCTCTGACTGTGAGAGC 3′, reverse 5′ GCATCACTGTGCTGCTGGGAC 3′; *Ube1L*, forward 5′CGAGTCAGGATGGATGAAG 3′, reverse 5′CAGTAGGTCCTCAGTGATG3′; *UbcM8*, forward 5′TGATGAAGCGTCAGGAACTG3′, reverse 5′CCTCTTCCGTTGCGTACTTC3′; *Bcl-X_L_*, forward 5′TGGAGTCAGTTTAGTGATGTCG3′, reverse 5′CCAGCAGAACCACACCAGCC3′; *Irf7*, forward 5′CAGCGAGTGCTGTTTGGAGAC3′, reverse 5′AAGTTCGTACACCTTATGCGG3′; 18S RNA, forward 5′CGCCGCTAGAGGTGAAATTCT3′, reverse 5′CAATCTCGGGTGGCTGAAC3′; *ß-Actin*, forward 5′GTGGGCCGCCCTAGGCACCA3′, reverse 5′CTCTTTGATGTCACGCACGATTTC3′. *Herc6* [47]: forward 5′GGCAGTTGGCTCTCAGCGGG3′, reverse 5′CTCTGCGGGGGCCTCCTGAT3′; HPRT: forward: GCTGGTGAAAAGGACCTC reverse: CACAGGACTAGACCTGC. Signal quantification was performed by scanning the gels images and analyzed with Image J software. Raw data were normalized to either 18S (Fig 1C) or ß-actin (Fig. 2 B) signals. Statistical analysis was performed using at least three independent experiments with GraphPrism as indicated below.

### Flow cytometry

Cultured erythroblasts or single cell suspensions of freshly isolated spleen or bone marrow were obtained from 8 to 12 weeks old mice and were stained with fluorochrome-conjugated monoclonal antibodies, all from BD Biosciences. 2.10^6^ cells were washed with PBS, 3% of FBS, 10 mM of sodium azide and incubated for 30 minutes at 4°C with phycoerythrin (PE)– Ter119 antibody and either fluorescein (FITC)– anti CD71 or biotin-anti CD71. After washing, the cells were incubated with (APC)-streptavidin, 7AAD and FITC-AnnexinV when indicated. AnnexinV staining was performed using FITC-AnnexinV Apoptosis Detection Kit (BD Biosciences), according to the manufacturer's recommendations.

Cells were analysed on a FACSCalibur cytometer (BD Biosciences) or sorted using a FACS-ARIA II cytometer (BD Biosciences). For sorting, DAPI was used instead of 7AAD to exclude dead cells. Data were analysed with FlowJo (Tree Star).

### Immunoprecipitation and western blot

Erythroblasts were washed twice in ice-cold PBS and lysed according to cell number and volume (1.10^6^ cells/10 µl) in RIPA buffer containing 1% aprotinin; 100 µg/ml phenylmethylsulfonyl fluoride; 10 µg/ml leupeptin, 50 nM NaF, 10 mMNaPi and 10 mM N-ethylmaleimide (NEM). The lysates were cleared by centrifugation at 16.000 xg for 20 min, at 4°C. Immunoprecipitation were carried out using 20 µl of anti FLAG M2-agarose beads/500 µl cell lysates (Sigma-Aldrich), for 1 to 2 hours on a rotating platform. After 3 washes with RIPA, immunoprecipitates were eluted with Laemmli sample buffer, samples boiled and analyzed by western blotting. Proteins were separated by SDS-PAGE and processed for western-blot analysis using the indicated rabbit polyclonal antibodies: anti-STAT5 C17 (Santa Cruz), anti-ISG15 (a generous gift from D.J. Lenschow, Washington University School of Medicine, St Louis, Missouri 63110, USA), anti-ERK2 (sc-154 Santa Cruz), anti-STAT1 (Cell signaling, 9172), anti- PLCγ (Cell signaling 2822), anti-Globin N19 (Santa Cruz), and monoclonals anti-PY694/699-STAT5 (Upstate Biotechnology), anti-Bcl-X_L_ (Transduction Laboratories), anti- Flag (F3165 Sigma), anti-actin (AC15, Sigma), anti-GAPDH (mAB374, Millipore). For western blot quantification, films were scanned, bands quantified using ImageJ software and raw data normalized to ß-actin signals. Statistical analysis was performed using at least three independent experiments with GraphPrism as indicated below.

### Statistical analyses

Statistical analyses were performed using GraphPad Prism. The student t test was used to calculate P values (two tailed). P values of 0,05 or less were indicated by one asterisk, P values of 0,01 or less by two asterisks and P values of 0,001 or less by three asterisks. Data were presented as mean values plus or minus SEM.
